# Structural genome variants of *Pseudomonas aeruginosa* clone C and PA14 strains

**DOI:** 10.3389/fmicb.2023.1095928

**Published:** 2023-03-13

**Authors:** Jens Klockgether, Marie-Madlen Pust, Colin F. Davenport, Boyke Bunk, Cathrin Spröer, Jörg Overmann, Burkhard Tümmler

**Affiliations:** ^1^Department of Pediatric Pneumology, Allergology and Neonatology, Hannover Medical School, Hanover, Germany; ^2^Leibniz Institute DSMZ – German Collection of Microorganisms and Cell Cultures, Braunschweig, Germany; ^3^German Center for Infection Research, Partner Site Hannover-Braunschweig, Braunschweig, Germany; ^4^German Center for Lung Research, Biomedical Research in Endstage and Obstructive Lung Disease (BREATH), Hannover Medical School, Hanover, Germany

**Keywords:** *Pseudomonas aeruginosa*, genome evolution, inversions, IS elements, long-read sequencing, recombination sequences

## Abstract

Plasticity of *Pseudomonas aeruginosa* chromosomes is mainly driven by an extended accessory genome that is shaped by insertion and deletion events. Further modification of the genome composition can be induced by chromosomal inversion events which lead to relocation of genes in the affected genomic DNA segments, modify the otherwise highly conserved core genome synteny and could even alter the location of the replication terminus. Although the genome of the first sequenced strain, PAO1, displayed such a large genomic inversion, knowledge on such recombination events in the *P. aeruginosa* population is limited. Several large inversions had been discovered in the late 1990s in cystic fibrosis isolates of the major clonal lineage C by physical genome mapping, and subsequent work on these examples led to the characterization of the DNA at the recombination breakpoints and a presumed recombination mechanism. Since then, the topic was barely addressed in spite of the compilation of thousands of *P. aeruginosa* genome sequences that are deposited in databases. Due to the use of second-generation sequencing, genome contig assembly had usually followed synteny blueprints provided by the existing reference genome sequences. Inversion detection was not feasible by these approaches, as the respective read lengths did not allow reliable resolution of sequence repeats that are typically found at the borders of inverted segments. In this study, we applied PacBio and MinION long-read sequencing to isolates of the mentioned clone C collection. Confirmation of inversions predicted from the physical mapping data demonstrated that unbiased sequence assembly of such read datasets allows the detection of genomic inversions and the resolution of the recombination breakpoint regions. Additional long-read sequencing of representatives of the other major clonal lineage, PA14, revealed large inversions in several isolates, from cystic fibrosis origin as well as from other sources. These findings indicated that inversion events are not restricted to strains from chronic infection background, but could be widespread in the *P. aeruginosa* population and contribute to genome plasticity. Moreover, the monitored examples emphasized the role of small mobile DNA units, such as IS elements or transposons, and accessory DNA elements in the inversion-related recombination processes.

## Introduction

The genome organization of bacteria has been analyzed in the past by genetic linkage, physical mapping, and sequencing (in this chronological order). With the advent of pulsed-field gel electrophoresis in the mid-1980s (Schwartz and Cantor, 1984), the construction of genetic and physical genome maps became feasible (Smith et al., 1987). The bacterial chromosome was cleaved with rare-cutting restriction endonucleases, the generated fragments were ordered to a genomic cleavage map and genes were assigned to the macrorestriction fragments by hybridization (Birren and Lai, 1996). Most initial studies focused on Enterobacteriaceae. Inter- and intraspecies genome comparison within the genus *Salmonella* revealed a phylogenetically conserved gene order, but also a highly plastic chromosomal organization characterized by insertions of non-homologous DNA and transpositions and inversions caused by homologous recombinations between the *rrn* operons that code for ribosomal RNA (Liu and Sanderson, 1995a,b, 1996). Likewise, sublines of the *Pseudomonas aeruginosa* reference strain PAO1 were shown to differ in their chromosomes by a mobile prophage and a large inversion between two *rrn* operons from each other (Klockgether et al., 2010). An inversion between two *rrn* operons was later also detected for the chromosome of *P. aeruginosa* NH57388A (Irvine et al., 2019).

The ubiquitous aquatic bacterium *P. aeruginosa* has a five-group population structure whereby about 98% of isolates belong either to the *exoS*-positive group 1 or to the *exoU*-positive group 2 (Freschi et al., 2019). The two most frequent *P. aeruginosa* clones are clone C for group 1 and clone PA14 for group 2 (Wiehlmann et al., 2015; Lee et al., 2020). Starting with a physical genome map of a *P. aeruginosa* clone C isolate from the airways of a patient with cystic fibrosis (CF) as reference (Schmidt et al., 1996), genomic cleavage maps of 21 clone C strains have been constructed for the restriction enzymes SpeI, PacI, SwaI, and I-CeuI by one- and two-dimensional pulsed-field gel electrophoresis (Römling et al., 1997a,1994b). Besides a variable strain-specific composition of DNA blocks in the accessory genome, large chromosomal inversions were noted in several strains collected from people with CF. These inversions were generated by the mobile IS elements ISPa20 (Kresse et al., 2006) or IS*6100* of a class 1 composite transposon (Kresse et al., 2003) whereby the latter had been inserted into a chromosomal copy of the integrative and conjugative element (ICE) pKLC102 (Klockgether et al., 2004, 2007). The inversions disrupted genes involved in important phenotypic traits such as LPS biosynthesis, twitching motility or DNA repair, respectively, suggesting that genome rearrangements are involved in clonal speciation and niche adaptation (Kresse et al., 2003).

The former analysis of inversion recombination breakpoints, i.e., the two loci displaying homologous sequences at which the DNA was actually cut and re-ligated after inverting the genome segment located in between, required a laborious and technically demanding combination of high-resolution physical mapping and breakpoint-spanning amplicon sequencing (Kresse et al., 2003, 2006). Third generation sequencing technologies now offer the opportunity to resolve genomic structural variants by the base (West et al., 2022). Hence, we examined the clonal genomic plasticity of *P. aeruginosa* by PacBio SMRT and nanopore long-read sequencing of strain panels of the major clones C and PA14 that had previously been studied by pulsed-field gel electrophoresis (Römling et al., 1997a,1994b) and short-read sequencing (Fischer et al., 2016), respectively. Thereby numerous genomic structural variants were identified in isolates from clinical and environmental habitats suggesting that a plastic chromosomal organization is characteristic for *P. aeruginosa.* Insertion and inversion breakpoints were typically located in repeat regions of the accessory genome (Klockgether et al., 2011).

## Materials and methods

### Bacterial strains

*Pseudomonas aeruginosa* isolates used in this work had already been analyzed in former studies. A collection of clone C isolates obtained from CF airways and from acute infection had been subjected to pulsed-field gel electrophoresis based genome mapping using restriction digests and marker hybridization approaches (Römling et al., 1997a,1994b). The results had indicated inverted genomic segments for eight isolates. Six of these isolates, C4, C8, C10, C12, C15, and C19 (see Table 1) were selected for long-read sequencing, genome assembly and inversion screening in order to verify the results deduced from the genome mapping.

**TABLE 1:** Isolates analyzed by long-read sequencing.

Isolate	Source	Date of isolation	Geographic origin
**Clone C**			
C4	CF airways	1988	Hannover, Germany
C8	CF airways	1992	Hannover, Germany
C10	CF airways	1987	Hannover, Germany
C12	CF airways	1989	Hannover, Germany
C15	CF airways	1987	Hannover, Germany
C19	CF airways	1989	Hannover, Germany
**Clone PA14**			
HCF202	CF airways	2002	Hannover, Germany
HCF324	CF airways	2003	Hannover, Germany
CF4	CF airways	2003	Liverpool, UK
K4	Keratitis	2003/2004	Bristol, UK
K9	Keratitis	2003/2004	Newcastle, UK
PT2	Environmental (river)	1992	Mülheim, Germany

A collection of PA14 strains had been analyzed for intraclonal diversity in a study (Fischer et al., 2016) using short-read sequencing. No hints for chromosomal inversions could be drawn from the data, but intraclonal phylogeny was assessed by screening for nucleotide substitutions between strains. For chromosomal inversion screening by long-read sequencing in clone PA14 strains, six isolates from this collection, HCF202, HCF324, CF4, K4, K9, and PT2 (Table 1) were selected that represented different branches of the phylogenetic tree and originated from different habitats.

### Bacterial growth and genomic DNA preparation

*Pseudomonas aeruginosa* isolates were grown in Luria-Broth at 37°C for 16 h under constant shaking, and the cells harvested by centrifugation (10 min, 5,500 × *g*). Genomic DNA was extracted by following the principles of a protocol optimized for Gram-negative bacteria (Ausubel et al., 1987-1995) including SDS-based cell lysis and subsequent nucleic acid purification by phenol-chloroform extraction. In order to minimize shearing and fragmentation of the genomic DNA and to generate long DNA templates for the sequencing, the protocol was adapted to preserve DNA integrity. Volume of buffers and solutions were increased, and instead of pipetting and vortexing, DNA containing solutions were carefully decanted and only moderately shaken. Harvested cells from 2 ml bacterial culture were resuspended in 1,800 μl lysis buffer (40 mM Tris-acetate, 20 mM Na-acetate, 1 mM EDTA, 1% SDS, pH 7.8) by gentle swilling. After 20 min incubation, 600 μl 5 M NaCl solution were added and cell debris was removed by centrifugation (45 min, 14,000 × *g*). The decanted supernatant was then supplied with 30 μl RNase A solution (10 μg/μl) and incubated for 15 min. The DNA was further purified by extraction steps using (a) phenol, (b) phenol/chloroform/isoamyl alcohol (25:24:1; v/v/v), and (c) chloroform/isoamyl alcohol (24:1; v/v). For each step, the DNA solution and the organic solvent were mixed. After centrifugation (30 min, 12,000 × *g*), the aqueous phase containing the DNA was either carefully decanted, or the organic phase was removed by slow pipetting. Due to frequent leftovers of organic and interphase material in the aqueous phase, extraction step (b) was usually repeated once or twice. Subsequently, DNA was precipitated from the aqueous phase by the addition of an equal amount of isopropanol, pelleted by centrifugation (30 min, 14,000 × *g*) and washed again in 70% ethanol. The air-dried DNA pellet was finally dissolved in 100 μl low TE-buffer (10 mM Tris, 1 mM EDTA, pH 7.5) for storage. During these last steps, liquids were removed again just by decanting to avoid any pipetting of the precipitated DNA.

### Library preparation and sequencing

PacBio-SMRT sequencing was done on the *RSII* platform at the Leibniz Institut DSMZ (Braunschweig, Germany) using one SMRT™ Cell (Pacific Biosciences) for each *P. aeruginosa* isolate. Long-read sequencing was also done in house with a MinION device and R9.4 flow cells [Oxford Nanopore Technologies (ONT)]. In MinION runs, one flow cell was utilized for parallel sequencing of genomic DNA of up to 12 *P. aeruginosa* strains.

SMRTbell™ template libraries were prepared according to the instructions of Pacific Biosciences, Menlo Park, CA, USA, following the “Procedure & Checklist – Greater Than 10 kb Template Preparation.” Briefly, for preparation of 15 kbp libraries 8 μg genomic DNA was sheared using g-tubes™ from Covaris, Woburn, MA, USA according to the manufacturer’s instructions. DNA was end-repaired and ligated overnight to hairpin adapters applying components from the “DNA/Polymerase Binding Kit P6” (Pacific Biosciences). Reactions were carried out according to the manufacturer’s instructions. BluePippin™ Size-Selection to greater than 7 kbp was performed according to the manufacturer’s instructions (Sage Science, Beverly, MA, USA). Conditions for annealing of sequencing primers and binding of polymerase to purified SMRTbell™ template were assessed with the Calculator in RS Remote (Pacific Biosciences). SMRT sequencing was carried out on the PacBio *RSII* (Pacific Biosciences) utilizing one 240 min movie for each strain.

For MinION sequencing, the library preparation protocol “Ligation sequencing gDNA – native barcoding” applying kits SQK-LSK109 and EXP-NBD104 (ONT) was followed. A total of 400 ng genomic DNA per strain were used as starting material. The MinION runs were continued for at least 8 h. Base calling and barcode demultiplexing was done with the Guppy toolkit provided by ONT. Read quality was inspected with FASTQC 0.11.9 (Andrews, 2010), and reads were trimmed with Porechop v0.2.4 (Wick et al., 2017) and Filtlong 0.2.0 (available at Github platform).^1^ Porechop was applied for the elimination of adapter sequences, Filtlong was used with a minimum read length value of 1,000 nucleotides and a minimum read quality value of 10 in order to remove the 10% of reads with the lowest average quality values from the assemblies.

### Genome assembly

Long read genome assembly was performed with the “RS_HGAP_Assembly.3” protocol included in SMRTPortal version 2.2.0. Hereby, the target genome size was set to 5 Mbp. In all cases, a single contig-sequence could be obtained for the bacterial chromosomes.

Assembly of genome sequences from the MinION datasets of clone C strains was done with the Canu v1.8 assembly pipeline (Koren et al., 2017). For clone PA14 datasets, the Flye assembler, version 2.7.1 (Kolmogorov et al., 2019), was used. Both tools had been developed for long read assemblies. Parallel use of both tools on some clone PA14 datasets produced comparative genome sequence scaffolds with slightly higher coverage values for the Flye outcome, therefore the Flye results were used in subsequent sequence comparisons. Both assembly tools were run with default parameters and estimated genome size values of 6.5 Mbp. Minimum coverage values were set to 30 in Canu assemblies or 50 in Flye assemblies, respectively.

### Inversion detection

The assembled genome sequences were screened for inverted areas and repeat sequences by comparison with *P. aeruginosa* reference genomes, in case of clone C strains with the strain NN2 sequence (accession no. NZ_LT883143.1), and in case of clone PA14 with the strain PA14 reference sequence (accession no. NC_008463.1). Comparisons were done with the blastn tool of the NCBI blast+ toolkit (Camacho et al., 2009), applying either the downloaded program package on own servers or the toolkit hosted at the Galaxy platform (Cock et al., 2015). Repeat areas flanking inverted genome segments were inspected further by comparison with sequences of known genomic islands and other mobile DNA of *P. aeruginosa* in order to characterize the presumed inversion breakpoints.

### Phylogenetic analyses

Phylogenetic trees were originally calculated for clone C and clone PA14 reference panels in a previous intraclonal diversity study (Fischer et al., 2016) in which SNPs were determined using short-read sequencing data. Phylogenetic trees from the resulting core genome sequences were calculated with the SplitsTree software (Huson and Bryant, 2006). As Fischer’s strain panel contained all PA14 isolates used in this study, but only one of the six clone C strains, genome distance analyses were performed for the latter to display the phylogeny within this group of strains. For this purpose, genome distances were calculated for the assembled sequences of strains C4, C8, C10, C12, C15, and C19 and the NN2 reference sequence using the Genome BLAST Distance Phylogeny approach (GBDP) (Meier-Kolthoff et al., 2013). Results were used for inferring a minimum evolution tree with FASTME 2.1.6.1 (Lefort et al., 2015). Branch lengths are scaled in terms of the GBDP distance formula d_5_. The numbers represent GBDP bootstrap support values >60% from 100 replicated calculations. Distance calculations and tree construction were done at the Type Strain Genome Server TYGS platform (Meyer-Kolthoff and Göker, 2019) hosted at the Leibniz Institute DSMZ-German Collection of Microorganisms and Cell Cultures. The genome distance results might contains errors, as the genome sequences taken from this study for the calculations were derived from long-range sequencing data only and do not represent highly curated sequences. Since complete sequences including the accessory genome parts were used, however, distance errors caused by long-read sequencing bias should be small compared to the inter-strain diversity caused by individually composed accessory genomes. Therefore, the genome distance-based tree shown in Figure 2C should display a good estimation of the phylogeny within the small clone C panel.

### Data availability

Sequence read files and genome assemblies have been submitted to the public domain of the European Nucleotide Archive (ENA), study accession no. PRJEB57216.

## Results

### Genome sequencing on SMRT and nanopore platforms

The genomes of panels of *P. aeruginosa* clone C and clone PA14 strains (see Table 1) were sequenced on nanopore platforms. For comparison of technologies, the genomic DNA of two clone C strains was also sequenced on the PacBio *RSII* platform. Genomic structural variants had previously been demonstrated in the selected clone C strains by the construction of macrorestriction maps (Römling et al., 1997a,1994b). Table 2 lists the read numbers of the individual data sets. Genome assembly yielded circular chromosomes with sizes between 6.762 and 6.896 Mbp for the six clone C strains and between 6.515 and 7.026 Mbp for the six clone PA14 strains. The variable genome sizes reflect the intraclonal diversity of the accessory genome (Klockgether et al., 2011). The larger variability of genome size of the clone PA14 strains compared to that of the clone C strains is consistent with the different range of habitats and geographic origin. The clone PA14 strains were collected from disease and environmental habitats in Germany and the UK whereas the clone C isolates were retrieved within a 3 years period from the airways of people with CF regularly seen at the Hannover CF clinic (Römling et al., 1994a).

**TABLE 2:** Read numbers of the analyzed long read datasets and assembled genome contig sizes.

Strain	Number of reads	Size of assembled genome contig (bp)
**Clone C**		
C4	121,517	6,847,143
C8	82,979	6,896,472
C10	53,976	6,876,971
C12 (PacBio)	49,284	6,852,156
C12 (MinION)	186,627	6,847,448
C15	96,651	6,761,907
C19 (PacBio)	48,506	6,904,617
C19 (MinION)	62,575	6,895,419
**Clone PA14**		
HCF202	39,520	6,718,939
HCF324	36,908	6,514,680
CF4	88,457	6,703,718
K4	39,582	6,858,544
K9	70,753	7,025,649
PT2	66,276	6,785,000

The circular genome contigs assembled from PacBio and MinION datasets differed by 4.7 kb (strain C12) and 9.2 kb (strain C19) from each other (Table 2) corresponding to relative differences in genome size of 0.6 and 1.0%, respectively. However, the genomic structural variants in strains C12 and C19 were resolved with matching sensitivity and specificity by the two sequencing platforms suggesting that long-read sequencing is a suitable approach to map structural variants with high resolution.

### Overview of inversions detected by long-read sequencing

**FIGURE 1:**
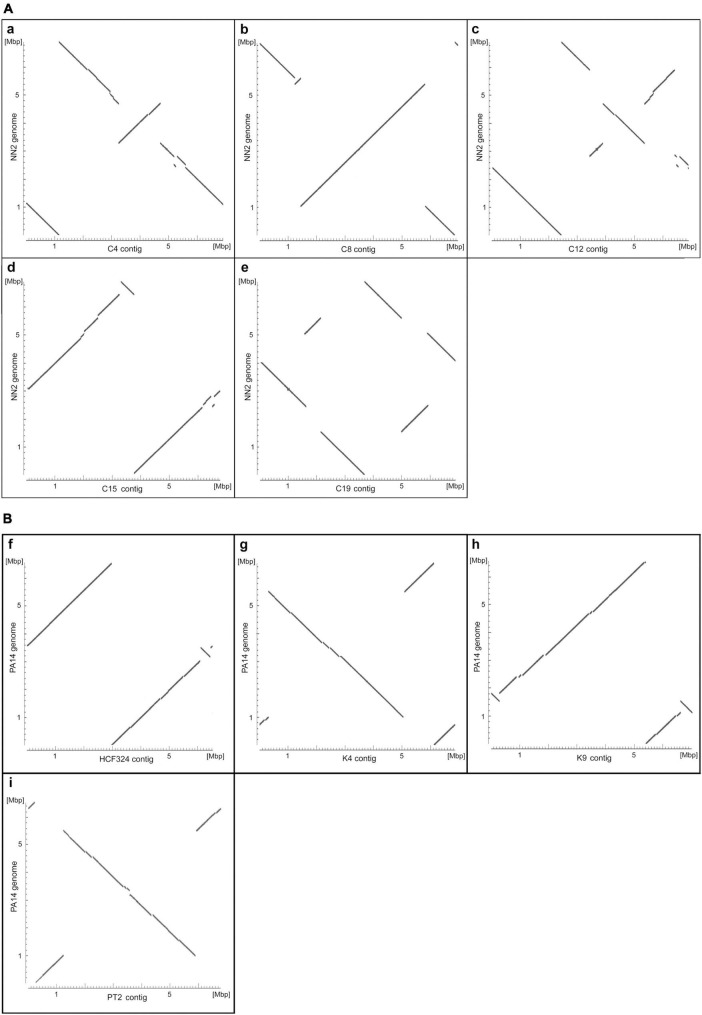
Alignment plots of clone C **(A)** and clone PA14 **(B)** genome assemblies. The plots display the highly similar sections of the assembled genome contigs of the clone C strains C4 (a), C8 (b), C12 (c), C15 (d), and C19 (e) and the strain NN2 sequence (NZ_LT883143.1) and of the clone PA14 isolates HCF324 (f), K4 (g), K9 (h), and PT2 (i) and the strain PA14 sequence (NC_008463.1). Sequence similarities were determined by pairwise blastn alignments of an assembled contig and the reference and appear as diagonal lines in the plots. Changes in the orientation of diagonal line segments indicate inverted DNA sections. Please note that, in contrast to the reference sequences, the sequence contigs of the other strains were not adjusted to start at the *oriC* region and could represent either the forward or the reverse DNA strand. Consequently, conserved regions between a contig and the reference sequence do not necessarily appear as diagonals starting at the bottom left corner of the plots.

Figure 1 shows alignment plots of clone C and clone PA14 genome assemblies. Sequence contigs were mapped to the genomes of the NN2 (Fischer et al., 2016) and PA14 (Lee et al., 2006) reference strains. Compared to the reference, insertions, deletions, and inversions were detected in five clone C (Figure 1A) and four clone PA14 strains (Figure 1B). The inversions in strains C4, C12, C15, and C19 previously resolved by low-resolution macrorestriction mapping (Römling et al., 1997b), were confirmed by long-read sequencing on the PacBio and nanopore platforms. In case of strain C8 a second small inversion was traced in addition to the already known large inversion. Conversely, an about 50 kbp large inversion in strain C10 that had been detected by pulsed-field mapping and later verified by sequencing of breakpoint-spanning amplicons (Kresse et al., 2003), was not observed in our C10 sequence contig representing a clone C strain by its clonal single nucleotide variants. Physical mapping and sequencing 20 years later had apparently been performed on non-matching DNA sources.

**FIGURE 2:**
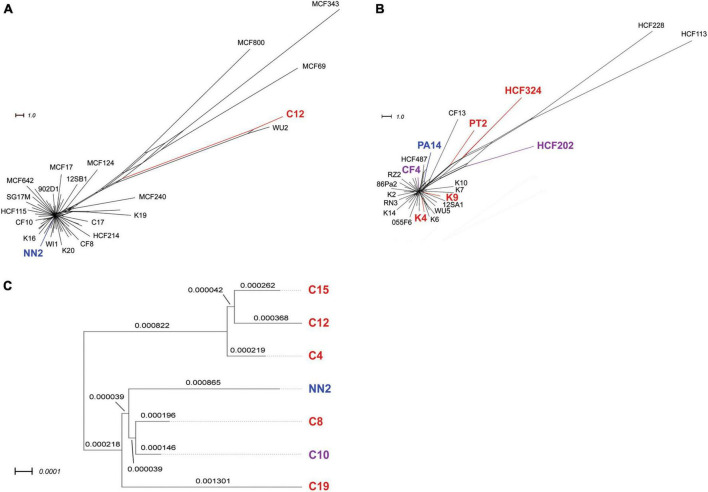
Phylogenetic trees of clone C and clone PA14 strain panels. The upper panels display phylogenetic trees for collections of 58 clone C strains **(A)** and 42 clone PA14 strains **(B)**, respectively. The trees were reconstructed from results on intraclonal diversity from our in-house report (Fischer et al., 2016) who had performed SNP based phylogeny analyses of the strains’ core genome sequences. All clone PA14 isolates used in the current study were chosen from this 42 strain panel, while only one of the six clone C strains screened for inversions (C12) was included the 58 strain panel analyzed in Fischer’s work. Therefore, genome distances were calculated for the six clone C strains and the reference strain NN2, and the results were displayed in a separate tree **(C)**. Branch lengths are given in terms of the applied GBDP distance formula (see section “Materials and methods”). Please note that MinION data-derived genome assemblies were used for these calculations, which could contain sequencing-based single nucleotide errors. Nevertheless, sequence diversity caused by strain-specific accessory genome composition should be reflected in these results. For all panels, strains included in the inversion screening in this study are indicated by either red (one or two inversions detected) or purple (no inversions) color. Reference sequences NN2 and PA14 for the two clonal lineages are shown in blue.

The genomes of the six clone PA14 strains had been examined previously by high-throughput short-read sequencing on a SOLiD 5500 XL platform (Fischer et al., 2016). Strains had been selected from our PA14 strain collection by the criterion of large intraclonal divergence within the phylogenetic tree. The SOLiD short read data sets had not provided any evidence for large-scale structural genome variants. However, as shown in Figure 1B, insertions, deletions and inversions were not rare, but actually seen in the majority of genomes investigated by long-read sequencing. Only the CF isolate CF4 shared the gross genome architecture with the PA14 reference. According to the analysis of this small clone PA14 strain panel, large inversions in *P. aeruginosa* are not confined to the habitat of CF airways as had been assumed during the pioneering physical mapping studies (Römling et al., 1997b; Kresse et al., 2003). Apart from occurrence during subculturing in the laboratory, as seen for the *P. aeruginosa* reference strain PAO1 (Stover et al., 2000), inversions can happen in all disease and environmental habitats of this aquatic organism.

Phylogenetic trees for clone C and clone PA14 strain panels based on short-read sequencing (Fischer et al., 2016) are provided in Figure 2. Strains analyzed for inversions in this study and the reference sequences are marked by colors to demonstrate the distribution of these strains in the larger panels from Fischer’s study. Different colors indicated the presence or absence of inversions in the tested strains.

The inversions of the different strains are described in detail in the following sections.

### Inversion events in single strains

The respective genomic inversion events that were observed in this study are described hereinafter in detail, accompanied by a figure for each strain displaying the supposed recombination breakpoints, flanking regions, etc. Comprehensive information is provided in Tables 3, 4. The figures show the affected genome regions for both the respective strain and the corresponding reference genome. The inverted segments are usually shown as blue hatched areas with the direction of the hatching lines representing the eventually inverted orientation of the DNA content in the two sequences. Accessory DNA elements that harbored the inferred recombination breakpoints or were located next to them are shown as gray blocks. If these breakpoints could be located in mobile elements such as IS elements or transposons, the respective sequences are marked in black. Purple bars represent ORFs from the reference genomes that are disrupted by the insertions of a DNA element providing the breakpoint sequence, a part of the ORF being then translocated during the subsequent inversion event. For strains with a second inversion event, the respective genome segment is marked by red hatching and potentially affected ORFs are displayed in gold. Please note that the size of genome segments and DNA elements in these figures could not be consistently displayed to scale.

**TABLE 3:** Inversions in clone C isolates.

Isolate	Source	Genome size (Mbp)	Inversion size^1^ (Mbp)	Breakpoint positions^2^	SpeI-fragments^3^	DNA elements	Comment^4^
C4	CF sputum (1988)	6.847	1.424	3,287,154ff.	SpA → SpF′	12.7 kbp transposon*	Symporter (PA2252) disrupted
				4,698.474–4,711,181	SpU → SpA″	12.7 kbp transposon	Part of PAGI-4/RGP7
C8	CF sputum (1992)	6.896	0.228	5,401,061ff.	SpL → SpI′	IS6100*	*warA* (PA4379) disrupted
				5,628,225–5,629,105	SpAB → SpI′	IS*6100*	IS-element in TNCP23/pKLC102
			4.569	1,037,982ff.	SpG → SpP′	IS6100*	PA4029 disrupted
				Before 5,606,940	SpAB → SpI′	No duplicated sequence	Part of TNCP23 incl. IS*6100* deleted
C12	CF throat swab (1989)	6.847	1.424	3,287,154ff.	SpA → SpF′	12.7 kbp transposon*	Symporter (PA2252) disrupted
				4,698.474–4,711,181	SpU → SpA″	12.7 kbp transposon	Part of PAGI-4/RGP7
			3.061	2,854,354–2,857,225	SpK → SpX′	2.9 kbp of RGP26	Duplication of part of RGP26
				Before 5,912,212	SpC → SpC′	2.9 kbp of RGP26*	Copy inserted at sRNA *prrF1*
C15	CF throat swab (1987)	6.762	6.393	55,623–55,629	SpAL → SpW′″	ISPa20*	PA0042 disrupted
				6,448,396–6,449,538	SpF → SpL′	ISPa20*	4.4 kb of RGP87 absent in C15
C19	CF throat swab (1989)	6.895	2.587	2,476,310–2,476,341	SpV → SpN′	32 bp within PAGI-2	Within ORF C65 of PAGI-2
				5,065,844–5,065,875	SpB → SpB″″	32 bp within NN2-RGP5	Within C65 homolog in NN2-RGP5
			4.053	1,552,188ff.	SpS → SpV′	IS6100*	*mutS* (PA3620) disrupted
				5,604,801–5,606,048	SpZ → SpS′	IS*6100*	1.2 kbp DNA of pKLC102 absent in C19

^1^Size of inverted area from left to right breakpoint region calculated from NN2 genome coordinates – could differ from the correct genome size if the accessory genomes of NN2 and the respective strain vary in this genomic region.

^2^Genome coordinates according to the NN2 reference genome (NZ_LT883143.1).

^3^SpeI fragments of the physical macrorestriction maps (Römling et al., 1997a). Table entries give the SpeI fragments containing the presumed recombination breakpoints of the inversion.

^4^For (truncated) core genome ORFs next to recombination breakpoints the number of the respective ortholog of reference strain PAO1 is given.

*Copy of respective element present in the inversion displaying strain, absent in the NN2 reference genome.

**TABLE 4:** Inversions in clone PA14 isolates.

Isolate	Source	Genome size (Mbp)	Inversion size^1^ (Mbp)	Breakpoint positions^2^	DNA elements	Comment^3^
HCF324	CF throat swab (2003)	6.515	0.326	3,174,603ff.	IS*6100* + 20.4 kbp spec. DNA	In PA14 no IS*6100* copies but RGP23 at left breakpoint; RGP23 in HCF324 in inverted area
				Before 3,500,638	IS*6100* + 25.5 kbp spec. DNA	ORF PA14_39320 (*rbsC*) disrupted by add. DNA
K4	Keratitis (2003/2004)	6.859	4.512	1,002,329ff.	7.3 kbp spec. DNA	No duplicated sequence, strain-spec. insertions at both breakpoints
				Before 5,514,000	52 kbp spec. DNA	
K9	Keratitis (2003/2004)	7.037^4^	0.692	1,120,794ff.	4.7 kbp spec. DNA	4.7 kbp fragment absent in PA14, doublets in K9
				Before 1,812,637	4.7 kbp spec. DNA (copy)	With traits of phage-like DNA
PT2	Water/river (1992)	6.785	4.512	1,002,329ff.	9.9 kbp spec. DNA	No duplicated sequence, strain-spec. insertions at both breakpoints similar to counterparts in K4
				Before 5,514,000	54.3 kbp spec. DNA	

^1^Size of inverted area calculated as part of PA14 genome from left to right breakpoint region – size could differ, if the accessory genomes of PA14 and the respective strain vary in this region.

^2^Genome coordinates according to the PA14 reference genome (NC_008463.1).

^3^For (truncated) core genome ORFs next to recombination breakpoints the number of the respective ortholog of reference strain PA14 is given.

^4^Genome size could be slightly different as assembled sequence contig could not be circularized.

### Clone C strains

#### Strain C4

**FIGURE 3:**
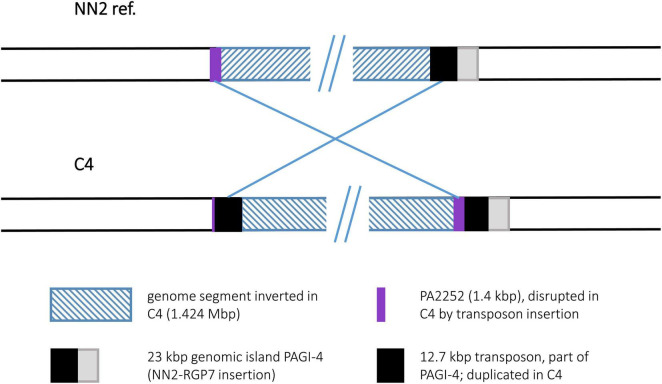
Genomic inversion in strain C4.

Comparison of the C4 sequence with the NN2 reference (Fischer et al., 2016) revealed a genomic inversion affecting a segment of approximately 1.424 Mbp (Figure 3). Identical copies of a 12.7 kbp transposon-like sequence were detected in C4 at both boundaries of this segment while the NN2 reference displayed only one copy at genome positions 4,698,474–4,711,181. This transposon sequence is part of the genomic island PAGI-4 (Klockgether et al., 2004). The second copy in the C4 sequence was found downstream of the NN2-genome position 3,287,154 thus splitting the sequence of the 1.45 kbp PA2252-orthologous ORF annotated as a Na-Ala-Gly symporter gene. In strain C4, only 80 bp of the 3′ end of this ORF were located at the left breakpoint region next to the transposon sequence while the other part was found at the other end of the inverted segment adjacent to the other transposon copy harboring the right breakpoint.

#### Strain C8

**FIGURE 4:**
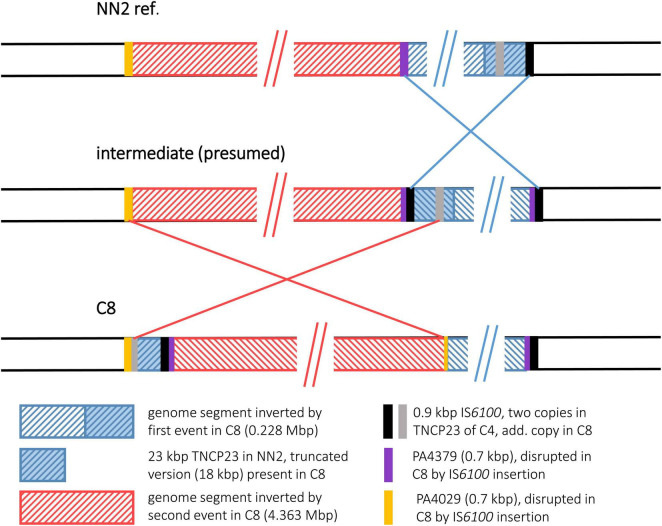
Genomic inversions in strain C8. For strain C8, two genomic inversions were observed that have affected overlapping genome segments. For better illustration of the recombination events that led to the observed DNA architecture in C8, a panel of the presumed intermediate was added that illustrates the DNA arrangement present after the first but before the second inversion event.

The C8 sequence displayed a more complex scenario of two inverted genome segments compared to the reference and a third segment with a distant location but original DNA orientation. We hypothesize that two inversion events led to this DNA arrangement that affected partially overlapping genome segments. Presumably, a first inversion involved a smaller segment of 0.228 Mbp. The recombination breakpoints of this event were located in copies of a 0.89 kbp IS*6100* insertion element, a small mobile DNA element that is found in the NN2 reference at the end of TNCP23, a 23 kbp transposon inserted in a bigger accessory element, the genomic island pKLC102 (Klockgether et al., 2004). In C8, an additional copy of IS*6100* had inserted into the middle of the 684 bp methyltransferase gene *warA*, a homolog of PA4379. Inversion of the DNA between these two IS*6100* loci resulted into a presumed intermediate DNA arrangement (Figure 4) before the genome organization of strain C8 was further reshaped by the second inversion event. This second event affected a larger segment of 4.569 Mbp. Part of this segment was approximately 18 kbp of TNCP23 that had already been affected by the first inversion and regained the original DNA orientation by this second inversion but was translocated to the other side of the large inverted area. At the left side of the second inversion, another IS*6100* sequence was located next to a 515 bp 3′ end fragment of a PA4029 homologous ORF indicating again IS*6100* as the recombination breakpoint sequence. The right flank, however, was marked by the leftover 151 bp of PA4029 only, but did not follow the remaining 5 kbp of TNCP23 of the NN2 reference, which would have harbored the counterpart of the IS*6100* sequence found at the left breakpoint. Apparently, this fragment with the second IS*6100* breakpoint of the larger inversion was deleted from the C8 genome in a subsequent recombination event. In other words, copies of IS*6100* sequences originally provided by the TNCP23 transposon repeatedly triggered inversions seen in strain C8.

#### Strain C12

**FIGURE 5:**
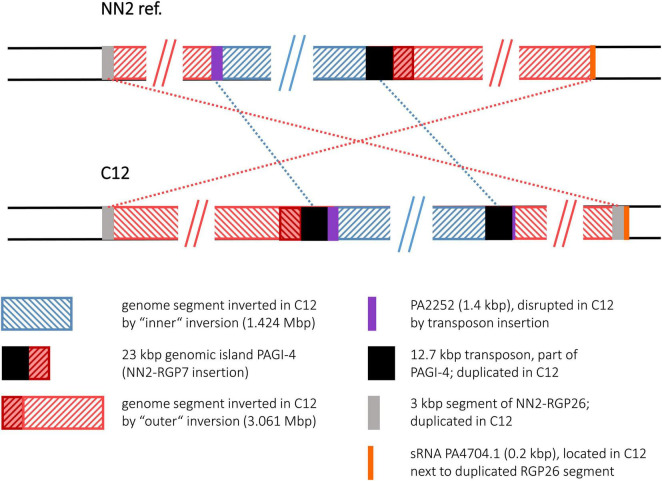
Genomic inversions in strain C12. This figure illustrates the two large inversions detected in the C12 sequence. As the genome segment of the smaller inversion was encompassed by the segment affected by the larger event, the inversions are shown as “inner” and “outer” events.

The C12 sequence displayed a scenario of two inverted and translocated genomic segments flanking a translocated DNA block in its original orientation. This arrangement was caused by two inversion events of which one (“outer inversion”) affected a larger segment completely encompassing the smaller segment affected by the other event (“inner inversion”). The sequence comparison with the NN2 reference gave no indications whether the inner or the outer inversion had occurred earlier. Both possibilities would have resulted in the observed C12 genome composition with the DNA affected by the smaller inversion being inverted twice and regaining the original orientation as seen in NN2 (Figure 5).

Interestingly, the inner inversion of strain C12 is identical with the inversion observed for strain C4. The same inverted 1.424 Mbp genome segment flanked by 12.7 kbp transposon sequence copies was detected, and as in strain C4, the PA2252 homologous ORF was split into an 80 bp and a translocated 1,366 bp fragment by the second transposon copy and the subsequent inversion. Including the area of the inner inversion, the outer inversion affected a total of 3.061 Mbp of the genome sequence leading to two segments (0.433 and 1.214 Mbp) of reverse DNA orientation compared to the NN2 reference. At the breakpoints of the outer inversion, no DNA of transposons or IS elements was detected. Instead, copies of a 2.87 kbp DNA block were found. This DNA is located at positions 2,854,354–2,857,225 of the NN2 sequence and part of an accessory DNA element (NN2-RGP26) but displayed no traits of integrase or transposase genes. This 2.87 kbp sequence was apparently duplicated in C12. The second copy had inserted next to the sRNA gene *prrF1* (PA4704.1) without disrupting it, subsequently triggering the outer inversion event in strain C12. The 2.87 kbp element presumably played the same role as the transposon or IS elements in the other inversions.

#### Strain C15

**FIGURE 6:**
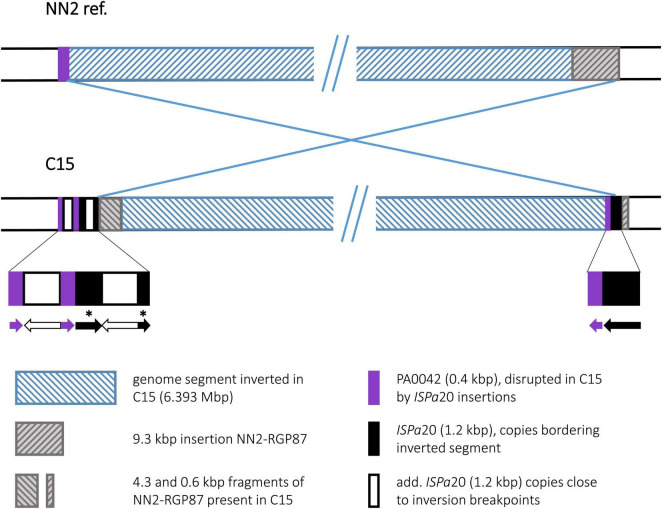
Genomic inversion in strain C15. The inversion observed in C15 is characterized by a complex DNA arrangement at the left breakpoint. Several copies of an ISPa20 element were detected. Two ISPa20 copies had presumably provided the inversion recombination sequences, while others inserted later at the left breakpoint region. In order to show the DNA arrangement in more detail, a third panel was added to the figure displaying the composition at the breakpoints. Arrows indicating the 5′ to 3′ direction in these sequences symbolize the DNA orientation of the different ISPa20 copies and ORF PA0042 fragments. Fragments of an ISPa20 copy disrupted by the insertion of an additional copy are marked by asterisks.

The C15 sequence displayed an inversion that affected a very large genomic segment of 6.393 Mbp (Figure 6). Recombination breakpoints were apparently located in ISPa20 insertion elements (Kresse et al., 2006) of which several copies were present in the C15 sequence whereas none was found in NN2. One ISPa20 copy was located at the right end of the inversion, following a 138 bp 3′ end fragment of ORF PA0042 and preceding a 0.6 kbp fragment of the accessory element NN2-RGP87. At the left end of the C15 inversion, a 4.3 kbp fragment of NN2-RGP87 is located next to ISPa20 DNA. Another 4.4 kbp of NN2-RGP87 were absent in C15 indicating a scenario in which ISPa20 copies had inserted in NN2-RGP87 and PA0042 of the C15 precursor sequence and provided the recombination sequence for the chromosomal inversion. The left breakpoint region was then affected by further recombination events. Due to these events, the NN2-RGP87 fragment that had been shifted to the left breakpoint region by the inversion was partially deleted. In addition, more copies of ISPa20 were inserted here, causing disruption of the 258 bp PA0042 remnant at this site and of the ISPa20 copy that had provided the recombination sequence. This ISPa20 copy is split into two fragments separated by an additional ISPa20 copy with reverse complementary DNA orientation (Figure 6, bottom panel).

#### Strain C19

**FIGURE 7:**
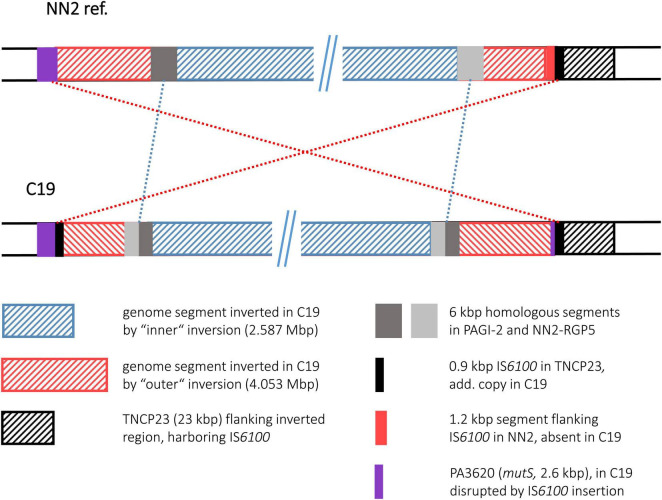
Genomic inversions in strain C19. This figure illustrates the two large inversions detected in in the C19 sequence. The inversions are shown as “inner” and “outer” events, as the genome segment of the smaller inversion was also part of the segment affected by the larger event.

Similar to C12, strain C19 displayed two inversions with an outer one completely encompassing the genomic segment of the inner one. The inner inversion in C19 affected a 2.587 Mbp segment whose ends were located in the large genomic islands PAGI-2 and NN2-RGP5. These related islands contain many highly similar DNA blocks, among them 6 kbp homologous DNA with 85–95% nucleotide sequence identity. In the NN2 reference, the 6 kbp block from PAGI-2 was located at the left side of the 2.587 Mbp segment and the corresponding 6 kbp block from NN2-RGP5 at the right side. In C19, both 6 kbp blocks appeared as chimeras, each composed of 3 kbp fragments from both PAGI-2 and NN2-RGP5 (Figure 7). In both cases, a 32 bp identical sequence was located between these halves. Therefore, these 32 bp copies (positions 2,476,310–2,476,341 and 5,065,844–5,065,875 in the NN2 reference) provided by different genomic islands presumably served as recombination sequence for the inner inversion. For the outer inversion, the recombination breakpoints were apparently provided by 0.87 kbp IS*6100* copies. As for the C4 and the C12 inversion, the copy at the right end was located in the TNCP23 transposon as it is found in the reference strain NN2. The additional copy in C19 that provided the left breakpoint sequence, had inserted in the 2.57 kbp ORF PA3620 encoding the mismatch repair protein MutS. Consequently, the ORF was disrupted and a 373 bp 5′ end fragment was translocated by the subsequent inversion event. The inversion was apparently accompanied by a small deletion event, as the C19 sequence lacked a 1.2 kbp fragment that was located next to the IS*6100* sequence in NN2 and should have been translocated to the left breakpoint region by the inversion. This fragment was part of the pKLC102 island sequence surrounding TNCP23 in the NN2 reference and contains a SpeI recognition sequence. The absence of the SpeI restriction site confirmed a particular feature of the C19 genome mapping results (Römling et al., 1997b) that had predicted two inversions in this strain the larger of which accompanied by a loss of a SpeI site.

### Clone PA14 strains

#### Strain HCF324

**FIGURE 8:**
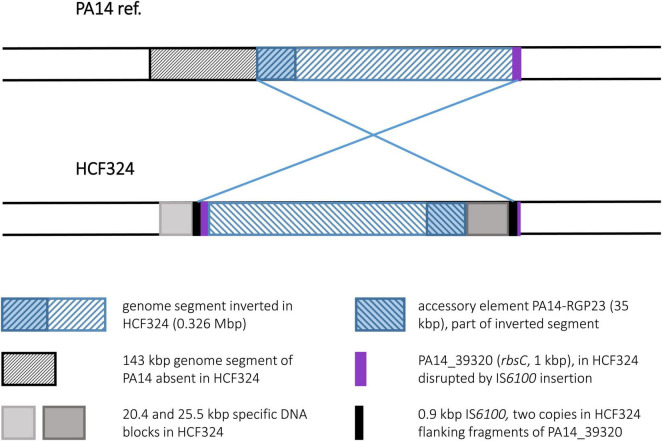
Genomic inversion in strain HCF324. This figure displays the genomic inversion detected in the HCF324 sequence and the different accessory DNA elements in strains PA14 and HCF324 in the breakpoint regions.

Comparison of the HCF324 sequence with the PA14 reference revealed the inversion of a 0.326 Mbp genome segment. As seen in some clone C strains, IS*6100* elements were identified of having provided the recombination sequences, as copies of this element were detected at both sides of the inverted DNA segment. IS*6100* was not present in the PA14 sequence at the respective sites, but in HCF324 one copy had inserted into the 1 kbp ORF *rbsC* (PA14_39320). Due to this disruption and the inversion, 185 bp of the ORF’s 5′ end were present in the right breakpoint area, while 814 bp of the 3′ end were found at the left breakpoint next to another IS*6100* copy. Prior to the inversion, this second IS*6100* copy had apparently inserted in an accessory DNA region. A total of 20.4 kbp accessory DNA with no counterpart in PA14 were found at the left breakpoint, outside of the inverted DNA segment. Another 25.5 kbp accessory DNA block was located close to the right breakpoint and could have been a part of the inverted DNA (Figure 8). If these accessory DNA blocks did not originate from insertion events that had followed the genomic inversion, they likely represent fragments of an accessory DNA element that was present in a HCF324 precursor in the left pre-inversion breakpoint area. The IS*6100* insertion then led to the disruption of this element and its partial translocation by the inversion. Similarly, the *rbsC* ORF was disrupted by the other IS*6100* copy. The left breakpoint region displayed further interesting features distinct from the PA14 reference. In PA14 the accessory element PA14-RGP23 consisting of transposon DNA and a fragment of the genomic island PAGI-1 (Liang et al., 2001) is located in this area. In HCF324 this DNA is part of the inverted segment. A total of 143 kbp of PA14 sequence upstream of the RGP23 site, however, were absent in HCF324. The region that harbored the left inversion breakpoint in HCF324 is apparently a region of high genomic plasticity.

#### Strain K4

**FIGURE 9:**
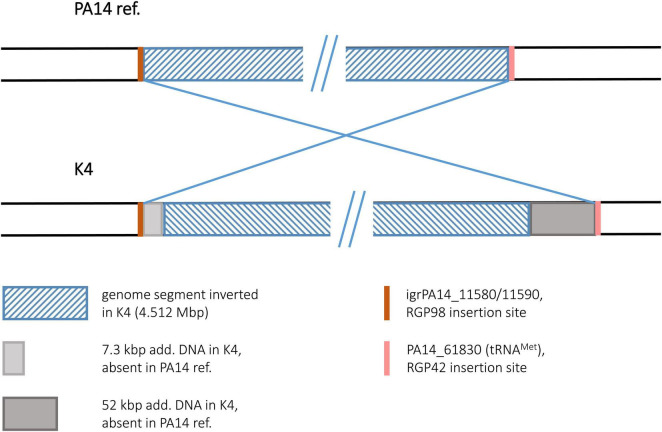
Genomic inversion in strain K4. This figure illustrates the genomic inversion detected in strain K4. No duplicated sequences were identified at the breakpoints in K4, instead accessory DNA was found in both regions. The insertion sites for these accessory elements are indicated by orange and pink bars.

The sequence of the keratitis isolate K4 displayed the inversion of a 4.512 Mbp genome segment. Interestingly, no duplicated sequences and no hints for transposon or IS element DNA were found at the termini of the inverted segment. Hence, no sequence features could be postulated as recombination sequences for this inversion event. At both ends of the inversion, however, accessory DNA blocks (7.3 and 52 kbp in size) were detected (Figure 9). Both blocks harbored DNA typical for mobile elements such as integrase or transposase genes and phage-like features although they did not fulfill all criteria of a mobile element. However, both blocks were positioned at a genomic site that had already been characterized as an insertion site for accessory DNA in other *P. aeruginosa* genomes. At the left, the accessory 7.3 kbp DNA were inserted in the RGP98 site (Sood et al., 2019), which is located in an intergenic region between ORFs PA14_11580 and PA14_11590. At the right, the 52 kbp accessory block was found at the RGP42 insertion site made up by the tRNA^Met^ gene PA14_61830 (Mathee et al., 2008). Similar to the clone C inversion examples, accessory DNA elements might have also played a role in this inversion in a clone PA14 strain, if these elements had already been present in a K4 precursor sequence prior to the inversion event.

#### Strain K9

**FIGURE 10:**
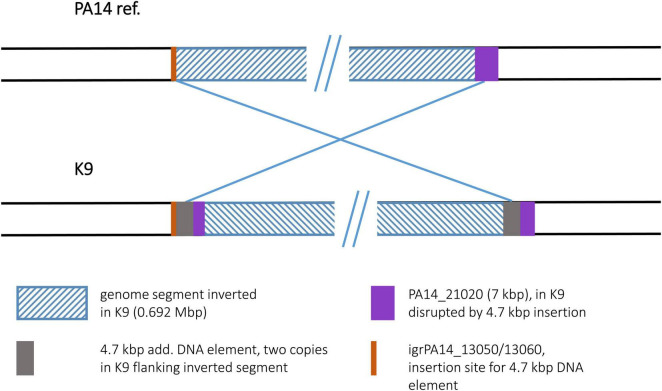
Genomic inversion in strain K9. The genomic inversion detected in strain K9 is flanked by two copies of a 4.7 kbp DNA element. At the left breakpoint region, this element had inserted into an intergenic region. This insertion site is illustrated by an orange bar.

For the K9 sequence, an inverted genome segment of 0.692 Mbp could be detected. This segment was flanked at both sides by copies of a 4.7 kbp DNA element that apparently harbored the recombination sequences for the inversion event (Figure 10). The 4.7 kbp element displayed phage-like DNA features and apparently played a role comparable to the transposon or IS element copies in other inversion examples. While absent in the PA14 reference, one copy of the 4.7 kbp element had inserted into a K9 precursor sequence in an intergenic region between ORFs PA14_13050 and PA14_13060. This site had not been described as a region of genome plasticity before. A second copy of the 4.7 kbp element had inserted into ORF PA14_21020 annotated as a non-ribosomal peptide synthase gene. The 7 kbp ORF was split into two fragments and the 3,023 bp 3′ end fragment became part of the inverted segment of the K9 sequence.

#### Strain PT2

**FIGURE 11:**
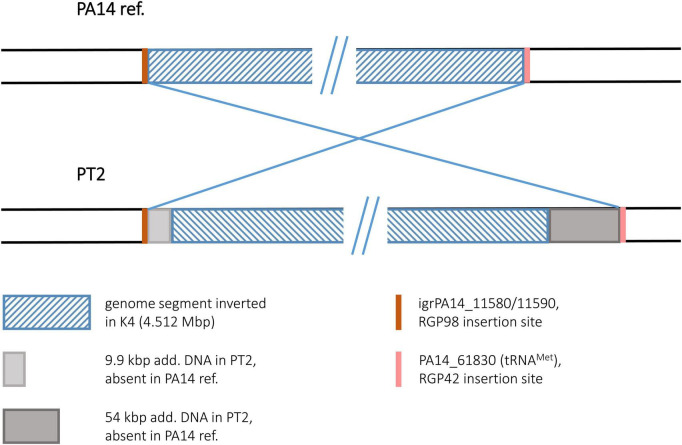
Genomic inversion in strain PT2. This figure illustrates the genomic inversion detected in the PT2 sequence. The same inversion event was also detected in strain K4.

To our surprise, the sequence of the environmental isolate PT2 displayed the identical inversion scenario that had been found for the keratitis isolate K4. For both sequences, the same inverted segment (4.512 Mbp) was detected, and as for K4, no duplicated sequences flanking this segment were found. Instead, accessory DNA elements flanked the boundaries. Like in K4, these accessory blocks in PT2 were located at the RGP98 and RGP42 insertion sites (Figure 11). Slight differences in the sizes of the blocks (K4: 7.3 and 52.0 kbp; PT2: 9.9 and 54.3 kbp) were caused by the presence of one or two small pieces of additional DNA in the PT2 versions of these accessory DNA elements, otherwise the harbored DNA was identical in the PT2 blocks and their respective K4 counterparts (>99.7% nucleotide identity). Therefore, both the K4 and the PT2 genomes likely were affected by the same genomic inversion event despite their different origins: K4 was collected 2003 from a patient with keratitis in Bristol, UK (Stewart et al., 2011) and PT2 was retrieved more than 10 years earlier from wastewater discharged into the river Ruhr in Mülheim, Germany (Römling et al., 1994a).

## Discussion

For a group of *P. aeruginosa* clone C isolates, genomic inversions had been determined by restriction-based genome mapping approaches (Römling et al., 1997a,1994b) and further analyzed by recombination breakpoint analysis (Kresse et al., 2003, 2006). We applied long-read genome sequencing to check whether the assembled contigs from this data would also display the same results and allow inversion screening by this method. PacBio sequencing of two isolates (C12 and C19) successfully confirmed the same genome segments as inverted as resolved by the mapping approaches. For isolate C19 that had been included in the combinatorial PCR-based breakpoint analysis approach, the arrangements of DNA from fragmented ORFs and IS elements at the breakpoint regions were also confirmed (Table 5). MinION sequencing of C12 and C19 displayed the same results as the PacBio approach and was applied to four additional clone C strains with existing genome maps. While for one isolate (C10) the predicted inversion was not found in the corresponding sequence assembly, the sequence for C4, C8, and C15 displayed the inversions and breakpoint DNA compositions that were predicted by the previous studies. In case of C8, even a second inversion event could be determined that had not been predicted by physical mapping (Römling et al., 1997a). Method-dependent limitations of tracing smaller genome arrangement events had likely prevented this finding in the former mapping approach.

**TABLE 5:** Comparison of inversion characteristics resolved by physical genome mapping (Römling et al., 1997a), breakpoint-spanning amplicons (Kresse et al., 2003, 2006), and long-read sequencing (this work).

Isolate/Feature	Physical mapping and breakpoint analysis	Long-read sequencing (PacBio)	Long-read sequencing (MinION)
**Strain C4**			
Inversion	1.25 Mbp	N/A	1.424 Mbp
**Strain C8**			
First inversion	Not detected	N/A	0.228 Mbp
Recombination breakpoints	Not detected	N/A	IS*6100*
Inversion	4.09 Mbp	N/A	4.569 Mbp
Recombination breakpoints	IS*6100*	N/A	IS*6100*
**Strain C10**			
Inversion	0.05 Mbp	N/A	Not detected
Recombination breakpoints	IS*6100*	N/A	Not detected
**Strain C12**			
“Inner” inversion	1.25 Mbp	1.424 Mbp	1.424 Mbp
“Outer” inversion	2.81 Mbp	3.061 Mbp	3.061 Mbp
**Strain C15**			
Inversion	5.89 Mbp	N/A	6.393 Mbp
Recombination breakpoints	ISPa20	N/A	ISPa20
**Strain C19**			
“Inner” inversion	2.45 Mbp	2.587 Mbp	2.587 Mbp
Recombination breakpoints	N/A	32 bp in homologous DNA of genomic islands	32 bp in homologous DNA of genomic islands
“Outer” inversion	3.93 Mbp	4.053 Mbp	4.053 Mbp
Recombination breakpoints	IS*6100*	IS*6100*	IS*6100*
Specific trait	Loss of SpeI site	1.2 kbp incl. SpeI site deleted	1.2 kbp incl. SpeI site deleted

Having confirmed the general applicability of long-read sequencing to discover genomic inversion events, we then used the less elaborate MinION sequencing approach to screen additional *P. aeruginosa* strains. While the analyzed clone C collection represented CF airway isolates retrieved from patients of related geographic origin and collected in the same clinical facility, isolates of more diverse origins were chosen. For that, we selected isolates from a clone PA14 strain collection from different clinical or environmental background. To our surprise, sequence contigs of four out of six isolates displayed inversion events, indicating that inversions are not limited to isolates from a chronic infection in an atypical habitat but could occur more frequently in the *P. aeruginosa* population than expected.

By applying high-throughput short-read sequencing technologies, the intra- and interclonal diversity of bacterial genomes has mainly been characterized by the nucleotide sequence diversity of the core genome and the variable composition of the accessory genome (Joseph and Read, 2010; Freschi et al., 2019). This work of the last years has resolved sequence diversity by the base but due to intrinsic technical constraints did not provide comprehensive information about genomic structural variants. Conversely, low-resolution physical mapping by pulsed-field electrophoresis techniques developed 40 years ago reliably identifies large-scale variants of genome organization caused by inversions, transpositions or duplications (Liu and Sanderson, 1996; Römling et al., 1997a,1994b). Insertions and deletions of 1 kbp or more are recognized by PFGE, but the high-resolution analysis of sequence variants, oligonucleotide repeats and gene order requires nucleotide sequencing that in case of complex repeat elements may need to be backed up by independent mapping approaches (Kresse et al., 2003). In principle, long-read sequencing should overcome these obstacles of comparative bacterial genomics and should reveal all variants true to scale and nucleotide identity.

The physical genome analysis of *P. aeruginosa* clone C isolates from people with CF seen concurrently at the same CF clinic is one of the few historical showcases of intraclonal genome comparisons based on PFGE mapping protocols (Römling et al., 1997b). Hence, having the original isolates still in our strain collection and the complete genome sequence of one isolate of this collection at hand, we decided to revisit the issue of structural genome variants of clone C CF isolates by the two current long-read sequencing technologies. Table 5 compares the outcome of the previous physical mapping approaches with that of SMRT and nanopore sequencing, respectively. Macrorestriction mapping and third-generation sequencing reliably identified the megabase-sized inversions in the C4, C8, C12, C15, and C19 genomes. However, in case of PFGE the low number of rare-cutter restriction sites and the non-linear dependence between fragment size and its migration velocity during PFGE inherently limited the accuracy of the assignment of the inversion breakpoints to a window of 10 kbp or more. Subsequent sequencing of breakpoint-spanning amplicons had identified the duplication – insertion events and the disrupted ORFs (Kresse et al., 2003, 2006), but this laborious approach could of course not detect any further changes out of the genomic segments targeted by PCR. Third-generation sequencing provided the whole picture of the complex genomic rearrangements in the five strains including two overlapping (C8) and two nested (C12 and C19) inversions, copy number variations of mobile elements (C15) and 1–5 kbp large insertions (C12) or deletions (C8, C15, and C19) flanking the inversion. PacBio and MinION sequencing was straightforward by protocol, but admittedly, the subsequent minute *in silico* analysis of the data sets took some time, albeit definitely many months less than Andreas Kresse’s two pioneering wet-lab studies (Kresse et al., 2003, 2006).

As the clone C reference strain NN2 and the examined clone C strains were supposed to be phylogenetically close, duplications, indels, inversions, and breakpoints were resolved with high resolution by comparison with the NN2 genome sequence. The structural variants of the clone PA14 panel could not be mapped with such a high precision because the isolates and the PA14 reference varied in their composition of the accessory genome in accordance with their divergent geographic origin, collection time, and habitat. Despite the different breadth of intraclonal genome diversity between the clone C and PA14 panels, genome plasticity emerged by a similar mode: the initial duplication of a mobile element was followed by the large chromosomal inversion. In other words, clone C and clone PA14 shared the genome organization of their structural variants, i.e., large chromosomal inversions carried a duplicated sequence at their boundaries. All genome rearrangements initiated in the accessory genome and terminated in either the accessory or the core genome whereby the terminus typically disrupted a gene.

The localization of breakpoints within the accessory genome might be the reason why large-scale genomic structural variants have not been documented in most bacterial genome projects that were based on high-throughput short-read sequencing. Breakpoints often reside within repeats and/or mobile regions of the accessory genome and thus escape notion when contigs of short reads are mapped on a reference genome for final assembly and ring closure. Interestingly, the physical mapping studies from the 1990s predominantly detected inversions between *rrn* operons (Liu and Sanderson, 1995a,b, 1996; Stover et al., 2000) but only occasionally recognized a breakpoint in the variable accessory genome. Likewise, within our current era of high-throughput sequencing Page et al. (2020) designed an *in silico* tool called *socru* that identifies all large-scale genome rearrangements that span an *rrn* operon. The tool resolves genome order by orientation of the *rrn* operons. Numerous rearrangements were detected in genomes with three or more *rrn* operons. Our study is in line with Page’s *in silico* finding that large chromosomal rearrangements are not rare. However, since the termini are often located in the flexible accessory genome and/or the inverted segment does not span an *rrn* operon, the genome order defined by the *rrn* landmarks will not be necessarily affected by the rearrangement.

By selecting clone PA14 isolates without any knowledge of their genomic architecture, we detected large inversions in four out of six examined strains. A technical bias of protocols and technology may be the reason why the frequency of genomic structural variants has been underestimated in the literature. As exemplified in this work, a wider application of long-read sequencing technologies may overcome our limited knowledge of large scale bacterial genome plasticity and its role in genome evolution in the real world (West et al., 2022). The frequency of inversion events observed in the few samples so far, up to two events per strain, is apparently lower by at least one order of magnitude compared to the dozens of smaller scale insertion and deletion events that promote plasticity and diversification of *P. aeruginosa* genomes (Mathee et al., 2008; Klockgether et al., 2011). Therefore, large inversions are likely a minor contributor to the flux of genome evolution, although each individual event alters the global genome architecture.

Besides their contribution to bacterial microevolution, large scale inversions could also play a role in adaptation processes, depending on the functional impact of such an event. The extent of such an impact, however, can only be assessed vaguely and could vary from case to case. In general, inversions might have global effects on replication and transcription processes, as the distance of the affected genomic regions to the origin of replication is changed. In addition, the inversion event can lead to a positional shift of the replication terminus, as seen for several examples described in this manuscript. Potential impacts, e.g., on transcriptome patterns could be presumed which might affect general fitness and stress adaptation capacity. We are not aware, however, of any data that yet support such a hypothesis. For the strains we have analyzed in this study, we did not observe any unusual growth behavior during culturing. Nevertheless, potential effects under certain stress conditions cannot be excluded.

More specific functional impact can be assumed if the inversion disrupts genes with known functions such as *rbsC*, encoding a ribose transporter component, or the mismatch repair gene *mutS.* Assuming a loss of function, the respective strain should display a corresponding phenotype, and for strain C19 hosting a disrupted *mutS* gene, Kresse et al. (2003) indeed detected a mutator phenotype. Alteration of gene functions could also occur if the disruptions do not directly affect coding sequences but promotor regions located in intergenic regions.

The list of published inversion events in *P. aeruginosa*, however, does not support the hypothesis of specific gene function loss as a driving force for genomic inversions during targeted adaptation processes. Instead, genomic inversions likely occur as random events in *P. aeruginosa* and preferentially target the accessory genome. Therefore, the frequency of these events probably depends on the clone- or even strain-specific accessory DNA composition and the presence and activity of mobile DNA elements as potential driving forces.

## Data availability statement

The sequencing data presented in this study can be found in an online repository. The data is deposited at the European Nucleotide Archive (ENA) (https://www.ebi.ac.uk), study accession number: PRJEB57216.

## Author contributions

JK conceived the study, collected and processed the bacterial isolates, performed sequencing on the nanopore platform, processed and evaluated the sequencing data, and designed the illustrations, and wrote the manuscript (draft and final form). M-MP set up the nanopore platform and performed library preparation and MinION sequencing. CFD set up the nanopore platform and processed the sequencing data. BB performed the SMRT sequencing and processed the PacBio sequencing data. CS performed the library preparation and SMRT sequencing. JO revised the manuscript draft. BT conceived the study and wrote the manuscript (draft and final form). All authors contributed to the article and approved the submitted version.

## Footnotes

1
https://www.github.com/rrwick/Filtlong
